# Trauma and loss in the Adult Attachment Interview: Situating the unresolved state of mind classification in disciplinary and social context

**DOI:** 10.1177/09526951221143645

**Published:** 2023-02-08

**Authors:** Lianne Bakkum, Carlo Schuengel, Sarah L. Foster, R. M. Pasco Fearon, Robbie Duschinsky

**Affiliations:** 2152 University of Cambridge, UK; 1190 Vrije Universiteit Amsterdam, The Netherlands; 1190 Vrije Universiteit Amsterdam, The Netherlands; 5995 Northumbria University, UK; 2152 University of Cambridge, UK; 2152 University of Cambridge, UK

**Keywords:** Adult Attachment Interview, attachment theory, loss, trauma, unresolved attachment

## Abstract

This article examines how ‘trauma’ has been conceptualised in the unresolved state of mind classification in the Adult Attachment Interview, introduced by Main and Hesse in 1990. The unresolved state of mind construct has been influential for three decades of research in developmental psychology. However, not much is known about how this measure of unresolved trauma was developed, and how it relates to other conceptualisations of trauma. We draw on previously unavailable manuscripts from Main and Hesse's personal archive, including various editions of unpublished coding manuals, and on Main–Bowlby correspondence from the John Bowlby Archive at the Wellcome Trust in London. This article traces the emergence of the unresolved state of mind classification, and examines the assumptions about trauma embedded in the construct. These assumptions are situated both in the immediate context of the work of Main and Hesse and in terms of wider discourses about trauma in the period. Our analysis considers how a particular form of trauma discourse entered into attachment research, and in doing so partly lost contact with wider disciplinary study of trauma.

## Introduction

Trauma has been conceptualised in different ways across time and context, including variously as discrete distressing events and as the consequences of such events. Modern conceptualisations of trauma include traumatic hysteria (Freud, 1966[1896]), war neurosis (Rivers, 1918), and post-traumatic stress disorder (PTSD; American Psychiatric Association, 1980). Trauma discourses change over time, and these changes may provide a lens on wider issues in the conceptualisation of the human mind, including memory and consciousness (Fassin and Rechtman, 2009; Hacking, 1994; Trembinski, 2011; Zajko, 1996).

One influential articulation of trauma discourse lies within the field of attachment research. In the late 1980s, the American psychologist Mary Main and her colleagues introduced the concept of an unresolved state of mind with respect to attachment as a category of the Adult Attachment Interview. The unresolved state of mind category was derived semi-inductively from confused or disrupted speech about loss or childhood abuse in adults’ autobiographical narratives. Main and colleagues proposed that adults with an unresolved state of mind had not adequately processed past loss or abuse and that these adults were still traumatised by memories of these experiences (e.g. Main and Hesse, 1990). The coding system for unresolved states of mind^1^ has been used for decades by researchers and clinicians.

Main and colleagues’ use of linguistics to investigate patterns of attachment has been described as a ‘revolutionary shift’ in attachment research (Van IJzendoorn and Bakermans-Kranenburg, 1997) and as the start of attachment research ‘Phase 2’ (Holmes, 2009). Their work has been influential for research in developmental psychology and psychopathology (Bakermans-Kranenburg and Van IJzendoorn, 2009; Verhage *et al*., 2016), and has informed psychotherapeutic practice, parenting interventions (e.g. Steele and Steele, 2008), and health policy (e.g. National Institute of Health and Care Excellence, 2015). However, there has yet to be any sustained examination of how ‘trauma’ has been conceptualised in the Adult Attachment Interview.

The only historical analysis of the unresolved state of mind construct, or indeed Main's work in general, has been by Duschinsky (2020). However, Duschinsky's account of the unresolved state of mind construct is brief and serves primarily to highlight the category as a pivotal concept for the history of attachment theory. The historiography of attachment theory has mostly focused on the life and work of an earlier generation of scholars before Main: John Bowlby (e.g. Van der Horst and Van der Veer, 2010; Van der Horst, Van der Veer, and Duschinsky, 2020) and Mary Ainsworth (e.g. Van Rosmalen, Van der Veer, and Van der Horst, 2015; Van Rosmalen, Van der Horst, and Van der Veer, 2016), the founders of attachment theory. This article contributes to historical research on the evolution of attachment theory after Ainsworth, by looking at the contributions of Main and her colleagues and placing these in a wider disciplinary and social context.

In their published writings, Main and colleagues have elaborated little on what they consider the meaning of an unresolved state of mind. A key obstacle to discussion of unresolved states of mind by other psychologists and historians of developmental science has been that the Adult Attachment Interview coding system has never been published; various editions have circulated in manuscript form only to attendees of accredited training institutes. In this article, we trace the emergence of the unresolved state of mind classification and examine assumptions about trauma embedded in the construct. This is facilitated by access to previously unavailable manuscripts from Main and Hesse's personal archive, including various editions of unpublished Adult Attachment Interview coding manuals. We also draw on Main–Bowlby correspondence from the John Bowlby Archive at the Wellcome Trust in London.

Our work also seeks to contribute to the history of conceptualisations of trauma. Frequently this literature has treated trauma discourses as if they circulate readily and isomorphically between domains. The case of the unresolved state of mind construct is of interest as an instance where a particular form of trauma discourse entered a relatively insular area of scientific practice; and as a result partly lost contact with wider trauma discourse and scientific study of trauma, while also having influence on health policy and professional practice.

This study identifies three notable, and historically contingent, assumptions about trauma in the work of Main and colleagues in introducing the unresolved state of mind classification. These assumptions were influenced by Bowlby's work on trauma and loss. However, we also consider the context of wider contemporary trauma discourses, such as PTSD and discourses about child abuse. Finally, we discuss the way that the unresolved state of mind construct became sequestered from wider disciplines of trauma research.

### Histories of attachment research

Attachment theory was founded by the British psychiatrist John Bowlby. Bowlby (1973) proposed that children's early experiences with their caregivers could shape cognition, emotion, and behaviour in later relationships. He suggested that separation experiences, including loss, could have detrimental effects on the mental health of children and adults. Historians of attachment theory have focused on the development of Bowlby's thinking, describing how experiences from Bowlby's early life (Van Dijken, 1998) and his studies and clinical work (Van der Horst and Van der Veer, 2009; Van Dijken *et al*., 1998) may have influenced his ideas.

The American Canadian psychologist Mary Ainsworth started working with Bowlby in the 1950s, and elaborated on his ideas by empirical work. Historians have regarded Ainsworth as the cofounder of attachment theory (Van Rosmalen, Van der Veer, and Van der Horst, 2015; Van Rosmalen, Van der Horst, and Van der Veer, 2016). One of Ainsworth's major contributions to attachment theory has been the Strange Situation procedure, a brief laboratory procedure that allows for observation of children's attachment behaviour during brief episodes of separation and reunion with the caregiver (Ainsworth *et al*., 1978). Three patterns of attachment behaviour were proposed: a group of infants who were visibly upset by the separation but adapted when the caregiver returned (Group B, ‘securely attached’), a group of infants showing little distress upon separation from their caregiver (Group A, ‘insecure-avoidant’), and a group of infants who were highly distressed throughout the procedure and were not easily soothed upon reunion with their caregiver (Group C, ‘insecure-ambivalent’). The origins of the Strange Situation procedure have been documented by Van Rosmalen, Van der Veer, and Van der Horst (2015).

With the aim to replicate and extend Ainsworth's findings, Mary Main started her own lab at the University of California in Berkeley in the early 1970s. Main completed her doctoral thesis under supervision of Ainsworth and was a close colleague of hers. Main and her colleagues at Berkeley recruited a sample of 189 parents from middle- and upper-middle-class backgrounds: the Berkeley Social Development Study. Observations of children's Strange Situation video recordings from this cohort and high-risk samples from other research labs led Main and Solomon (1986, 1990) to introduce the infant disorganised/disoriented (D) classification. Infants classified with disorganised attachment were earlier found unclassifiable with Ainsworth's A/B/C system. The emergence of the infant disorganised attachment classification has been traced by Duschinsky and colleagues, who described how this category has been shaped by contemporary conceptualisations of madness and ‘breakdown of behaviour’ (Duschinsky, 2015; Reijman, Foster, and Duschinsky, 2018).

## The Adult Attachment Interview

The Adult Attachment Interview was developed in the early 1980s, as part of Main and colleagues’ Berkeley Social Development Study. The families in this study participated in the Strange Situation when the children were 12 months old (with mother) and 18 months old (with father). A subset of these families were invited back to the laboratory for additional assessments when the children were six years old, in 1982. One of these assessments involved the parents taking part in an interview about their early attachment-related experiences: the Adult Attachment Interview. This semi-structured interview was developed by Main and her graduate students Carol George and Nancy Kaplan as part of their thesis projects.^2^ In the first question of the interview, participants were asked to describe the relationship with their parents as a young child and to choose five adjectives to describe the relationship with each parent. Other key questions addressed early separations from parents, experiences of rejection, and why participants thought their parents behaved as they did. Participants were also asked about experiences of loss of important persons such as parents in childhood and adulthood.^3^ Later versions of the interview protocol included questions about threatening behaviour by parents, experiences of abuse within the family, and potentially traumatic experiences other than loss and abuse.^4^

The coding system for the Adult Attachment Interview was developed based on a ‘guess and uncover’ method. This approach was described by Main and Cassidy (1988) and explained in more detail by Duschinsky (2020). Ruth Goldwyn, a research assistant in Main's lab, was given the task to study parents’ Adult Attachment Interview transcripts and try to guess the probable infant Strange Situation classifications:

Developing the system involved moving (blind) through each transcript in the development sample, and in each instance using feedback (‘correct’ or ‘incorrect’) with respect to the infant's attachment classification to that adult) to refine and to further develop the rule system. This is a slow-moving but highly profitable method of rule development, and it was used in the creation of every succeeding system.^5^

Through this inductive process, distinct patterns of adult discourse were identified.

Main and colleagues theorised that patterns of behaviour by children in the Strange Situation and by adults in presenting their autobiographical accounts in the Adult Attachment Interview could both be conceptualised as ways of organising attention in relation to attachment-related information. Later, Main would refer to differences in adults’ organisation of attention in relation to attachment-relevant information as ‘states of mind with respect to attachment’ (e.g. Main and Hesse, 1990). Main, Kaplan, and Cassidy (1985: 78–90) emphasised that ‘simply asking adults to verbalize their concepts of relationships’ would not work, because the actual ‘state of mind’ towards attachment could be different than what the adult might express in the interview in terms of content. Rather, coders of the Adult Attachment Interview examine the entire narrative for inconsistencies and contradictions, in order to explore the allocation and coherence of attention to attachment-relevant information.

A first group of parents identified by Main and Goldwyn were categorised as ‘autonomous/secure’ (F). These parents discussed attachment relationships with relative objectivity and ease, seemed to value attachment relationships, and were able to reflect on the influence of early attachments on their adult personality, regardless of whether past experiences were positive or negative. These parents frequently had infants who displayed secure attachment behaviour in the Strange Situation, five years earlier. One group of ‘insecure’ parents dismissed difficulties in early attachment relationships and regarded these as having little value or influence on their life (later termed ‘dismissing’ or ‘D’). According to Main and Goldwyn, dismissing speakers directed their attention away from (unfavourable) childhood memories; a pattern analogous to the behaviour of infants classified as insecure-avoidant in the Strange Situation. These infants showed little distress upon separation from the parent and actively directed their attention away from the parent on reunion. Another group of insecure parents seemed ‘preoccupied with dependency’ on their parents and ‘still actively struggled to please them’ (later termed ‘preoccupied’ or ‘E’). Preoccupied speakers were identified to correspond to infants classified as insecure-ambivalent in the Strange Situation. These infants seemed highly distressed and sometimes fearful throughout the procedure, constantly directing their attention towards the parent and unable to focus on the environment (Main, Kaplan, and Cassidy, 1985: 91).

### Unresolved loss

Main and Goldwyn observed that some interview transcripts of parents who had lost an attachment figure (a parent or other familiar caregiver) before adulthood showed incoherent and disrupted discourse across the interview. These individuals were frequently the parents of infants who were earlier found unclassifiable in the Strange Situation procedure. The unclassifiable infants would later be referred to as ‘disorganized/disoriented’ (D; Main and Solomon, 1986, 1990). Main and Goldwyn discovered that the presence of loss experiences was less predictive of infant disorganised attachment than how these events were narrated.

These observations laid the basis of an additional Adult Attachment Interview classification. Main, together with her husband and collaborator, Hesse, and a research assistant, Anitra DeMoss, developed a scale for identifying lack of resolution of mourning of attachment figures lost through death.^6^ The scale was based on the assumption that ‘cognitive disorientation and disorganisation’ were primary signs of lack of resolution of mourning, and that ‘irrationality of thought process with respect to a lost figure can be observed in speech’. A prominent example of lack of resolution of mourning was indications of disbelief that the person is dead. Other examples were irrational feelings of having caused the death of a loved one, discussion of the loss with an unusual attention to detail, and indications of confusion between the dead person and the self. In addition, Main described examples of extreme behavioural responses to loss in the past, such as a suicide attempt. Though these reports were considered rare, they were nonetheless included in the scale as indices of lack of resolution.^7^

Ratings on a 1–9 scale for lack of resolution of mourning were assigned based upon careful examination of the interview transcript, focusing on the speaker's description of the relationship with the lost figure, discussion of events surrounding the loss (such as the funeral), and reflection on how the loss might have affected the speaker. Ratings of 1 were assigned to speakers showing no signs of ‘disorganization or disorientation’, and ratings of 9 would apply to speakers who showed ‘definite disorganization, disorientation or evidence of irrational thought processes regarding a loss’. The description of the highest end of the scale includes mention of ‘traumatic loss’, which appears to be the first reference to trauma in the Adult Attachment Interview coding system.^8^

Main and colleagues came to refer to indices of lack of resolution of mourning as ‘lapses’ in the monitoring of reasoning, discourse, or behaviour. The term ‘lapse’ was first mentioned in a 1987 draft of the lack of resolution of mourning scale and was frequently used in later versions of the scale. Main's use of the language of ‘lapse’ to characterise indices of lack of resolution is interesting. Lack of resolution of mourning was intended to predict and correspond to the infant disorganised attachment classification. However, infant disorganisation was characterised by Main in the 1980s as an ‘interruption’ or ‘conflict’ of attachment pattern. By contrast, lack of resolution was characterised as a ‘lapse’ in state of mind regarding attachment. The term ‘lapse’ implies much more a falling away from a given state, whereas interruption or conflict imply the disruption of a state by internal or external forces. This difference in terminology suggests an initial lack of clarity about whether an identical, similar, or merely analogous psychological process was taking place for infants in the Strange Situation and adults in the Adult Attachment Interview. It was not clear to Main how this question might be tested empirically. However, her personal suspicion was that it was an identical process in lack of resolution and in disorganised attachment: both unresolved states of mind and infant disorganised attachment were later conceptualised as ‘lapses in working memory’ (Main and Hesse, 1992). On the basis of this supposition, from 1990, the lack of resolution of mourning scale was renamed ‘unresolved/disorganized/disoriented states of mind with respect to experiences of loss’.

Main and Hesse (1990) reported on the relation between parents’ unresolved loss and infant disorganised attachment in the Berkeley sample. The empirical association in this sample was strong: of the 12 mothers classified as unresolved, 11 had infants classified with disorganised attachment (later studies did not nearly find equally strong effect size estimates; Verhage *et al*., 2016). Main and Hesse hypothesised that the link between parents’ unresolved loss and infant disorganised attachment would be mediated by parents’ frightening/frightened behaviour in the presence of their infant. Informal observations of frightening/frightened parental behaviour led Main and Hesse to suggest that parents with an unresolved state of mind were still frightened or traumatised by past loss or abuse. They proposed that these parents’ frightening/frightened states were provoked by alarming memories of loss or abuse, leading to behaviours that could appear frightening to the infant, such as unusual vocal or movement patterns. However, the idea that (most) unresolved discourse was based on underlying frightening/frightened states involving alarming memories was not empirically tested. Main and Hesse (1990: 174–5) acknowledged that they were ‘not able to examine this issue in a satisfactory way on the basis of our present sample’ and stated that their informal observations of parental behaviour ‘tend to provide support for our hypothesis that the parent of the *D* infant may be frightening or frightened’. This apparently deductive inference resulted in an encompassing theoretical model of the association between parents’ unresolved states of mind and infant disorganised attachment, supported by concrete examples of frightening/frightened parental behaviour. Still missing, however, was a clear set of proposals about what unresolved discourse actually meant, as well as direct evidence of its inferred underlying mechanisms.

The assumption that unresolved states of mind and disorganised attachment would be underpinned by similar mechanisms of fear may have contributed to the appeal of the language of ‘transmission’ by attachment researchers when investigating empirical associations between the Adult Attachment Interview and Strange Situation (Van IJzendoorn, 1992, 1995). The assumption of intergenerational transmission of unresolved states of mind, with activated alarm as a potential underlying mechanism, has been influential for subsequent decades of research in the attachment field (Madigan *et al*., 2006; Verhage *et al*., 2016).

### Unresolved abuse

In the late 1980s, Main and colleagues became interested in unresolved abuse after seeing new interview transcripts collected by clinicians participating in training institutes for coding the Adult Attachment Interview (Duschinsky, 2020: 303). Main and colleagues discovered that lapses in the monitoring of reasoning and discourse also appeared surrounding discussions about childhood physical and sexual abuse by attachment figures, and that the presence of these indices was associated with infant disorganised attachment.^9^

Initially, coders were instructed to use the indices from the unresolved loss scale to mark unresolved abuse by attachment figures.^10^ Main finished a discrete version of a scale for coding unresolved abuse in 1991.^11^ As with unresolved loss, unresolved abuse was coded based on disrupted speech about abuse, and not on the basis of the abuse experience itself. However, the first step in coding unresolved abuse was to establish that any abuse experience described by the interviewee was ‘traumatic’ according to the standards of the Adult Attachment Interview coding system. According to the scale, when the speaker does not directly discuss a specific, potentially abusive experience, unresolved abuse may not be coded. Experiences that qualified as abuse were those that Main considered ‘overwhelmingly frightening’ to the child, such as hitting that leaves marks, being in pain after being badly hit, being locked in a closet as a punishment, and experiences of sexual abuse. Other experiences that qualified as frightening parental behaviour were threats to harm or kill the child and suicide attempts in the presence of the child. Excluded were parental behaviours that were perceived by the coder as distressing but not overwhelmingly frightening. Main noted that there might be cultural influences on what would be considered overwhelmingly frightened abuse: ‘In some sub-cultures spanking with the belt or a switch is expected in most families in the neighborhood, is seen by children as a natural form of discipline or punishment, and may therefore *not* be overwhelmingly frightening’.^12^

The exemplary indicator of unresolved abuse was ‘unsuccessful denial of the occurrence, nature, or intensity of the abusive experience’, which paralleled the ‘indications of disbelief that the person is dead’ lapse from the unresolved loss scale. Other indices of unresolved abuse were feelings of having caused one's own abuse, psychologically confused statements about abuse, disoriented speech, and fears of being possessed by the abusive figure. In addition, and indicative of Main and colleagues’ view that unresolved loss and abuse were functionally similar, coders were instructed to use indices from the unresolved loss scale to mark any unresolved abuse, and vice versa. From their perspective, what loss and abuse had in common were experiences of attachment figures that might make thinking about them alarming and overwhelming, causing both the aversion and the intensification of attention. However, there was an important difference between loss versus abuse experiences regarding the kind of person involved. In the interview, individuals were asked if they had lost any close or important persons, such as parents, in childhood or adulthood. Loss experiences could then be coded as unresolved for any deceased individual mentioned by the interviewee (i.e. attachment figures and other individuals, such as distant relatives). However, abuse could be coded as unresolved only if the perpetrator was an attachment figure, such a parent or another important caregiver. The nature of this discrepancy has not been clarified in the coding system.

Similar to unresolved loss, overall ratings of unresolved traumatic abuse were given on a 1–9 rating scale. When coding an Adult Attachment Interview, Main and colleagues advised that the scales for unresolved loss and unresolved abuse should be combined into one classification of unresolved/disorganised/disoriented state of mind. In the current version of the coding manual, the unresolved state of mind classification is assigned based on a rating of 5 or more on either the unresolved loss or the unresolved abuse scale, or both.^13^

In among the technical detail of the unresolved state of mind coding system are three important underpinning assumptions:

1. Bereavement is functionally identical to abuse as a kind of trauma when not psychologically reconciled.
2. Traumatic abuse experiences are defined by child physical and sexual abuse by attachment figures.
3. Lapses in adult discourse about unresolved bereavement or abuse experiences are underpinned by the same process as disruptions of attachment behaviour shown by young children.

Main did not appear to regard these as remarkable stances or even consider whether or how they might be testable. No argument is presented for them: they are taken for granted in her writing, and likewise have been accepted as hypotheses by Main's contemporaries and subsequent generations of attachment researchers. Yet all three assumptions can be regarded as contingent, shaped by the historical context of the formation of the unresolved state of mind construct.

### The influence of Bowlby: Segregated systems and defensive exclusion

A fundamental proximal influence on these assumptions was John Bowlby. Bowlby had originally wanted to focus his research on childhood physical and sexual abuse, having seen the influence of these experiences in his clinical work with children and families. However, he concluded that the topics were too controversial and that available scientific methods for assessing physical and sexual abuse were underdeveloped. Therefore, he decided to focus on loss rather than abuse.^14^ Shortly before Main and colleagues began their work on the Adult Attachment Interview, Bowlby published the book *Loss* (1980), bringing together his decades of research on the topic.

Drawing on cognitive information processing theory, Bowlby (1980) suggested a framework of ‘principal systems’ that existed within individuals. Examples included systems for organising behaviour for sex, friendly affiliation, eating, aggression, dominance, learning – and, of special interest to him, systems for care-seeking and caregiving. According to Bowlby, principal systems contained perceptual information, such as thoughts, feelings, and memories. In most individuals, these systems were unified and communicated freely with one another. However in some people, these systems were not unified, but segregated: one system might have access to one source or interpretation of information, and another system might have access to other information, and there would be restricted communication between the systems. These systems would then ‘differ in regard to what each perceived and how each interpreted and appraised events’ (ibid.: 63–4). According to Bowlby, segregation of systems was a defensive process that could be evoked by loss. He illustrated this with an example of pathological mourning in adults: ‘One system is oriented towards the lost object, longs for it, strives to recover it, and reproaches it for its desertion; the other system recognises its loss and organises behaviour on that basis.’^15^

Bowlby used the term ‘selective exclusion’ to refer to attentional processes that filtered incoming information from being further processed in consciousness. He referred to selective exclusion as an ‘integral and ubiquitous part of the action of the CNS [central nervous system]’, which was ‘proceeding every minute of our lives’.^16^ Bowlby believed that in most cases, selective exclusion was useful and adaptive. For instance, it was helpful to be able to filter unwelcome stimuli or memories when concentrating on a task. But selective exclusion might become maladaptive when it was long-term or persistent. This is what Bowlby called ‘defensive exclusion’. In the context of responses following loss, defensive exclusion could lead to pathological mourning:

Consider, for example, the processes that direct attention and activity away from painful thoughts and reminders and towards neutral or pleasant ones. When such processes take control only episodically they are likely to be fully compatible with health. When, by contrast, they become rigidly established they lead to a prolonged inhibition of all the usual responses to loss. (Bowlby, 1980: 139–40)

Bowlby anticipated that segregated systems and defensive exclusion could disrupt the capacity of an individual both to orient towards loved ones – in reality or in thought – and to achieve support and comfort. He termed this the ‘disorganisation’ and ‘disorientation’ of thoughts and feelings, and characterised clinical cases in which such disruptions could be seen in patients’ behaviour or their narratives in therapy. In his view, one of the central functions of therapy with such patients was to help reduce defensive exclusion of phenomena in the world, and reduce the segregation of mental systems. He anticipated this process would help the patient achieve reorganisation and reorientation to their life and enrich its possibilities.

In Bowlby's account, loss and abuse could cause both segregated systems and defensive exclusion. These processes could just as readily occur in children as in adults. Indeed, Bowlby's account of segregated systems and defensive exclusion was first developed as an attempt to provide an alternative to the ideas of Melanie Klein, while fully maintaining her assumption that grieving processes in infants were fundamentally the same as those in adults.^17^

Main was deeply immersed in Bowlby's writings on loss, and travelled to London to discuss them with him in 1978.^18^ Yet Bowlby's influence on her thinking about lack of resolution of mourning appears to have been tacit. One reason may have been that the account of segregated systems and defensive exclusion is exceptionally compressed in *Loss*. Only a few attachment researchers have ever subsequently discussed these concepts, primarily Inge Bretherton (2005) and Carol George (Solomon and George, 2011). It is telling that their treatments differ vastly from one another, and that Bretherton expresses hesitancy about whether she fully grasped Bowlby's meaning. Only a very close reading of *Loss* would make the concepts salient. In 1986, with the lack of resolution of mourning scale already developed, Main would write to Bowlby that

I have been re-reading your work on loss with the amazement I always experience in re-reading your work.… In developing the scale for lack of resolution of mourning we worked and re-worked our development cases, trying to determine the overriding feature. Disorganisation and disorientation of thought-processes or in apparent feeling seemed the best descriptor for what we were scaling. I then found myself amazed to read your description of recovery from loss in terms of re-organisation and re-orientation.^19^

As Main acknowledged, this was clearly an important influence, and it appears in tacit form across the coding manual, even if she did not use the terms *segregated systems* or *defensive exclusion*. For instance, ‘indications of disbelief that a person is dead’ is characterised by Main as a disconnect between two experiences of reality, one in which a loved one has died, and another, deactivated system storing the memory that the person is still alive. When the loss is discussed in the Adult Attachment Interview, the deactivated system may be activated briefly, resulting in a slip of the tongue to the present tense, without the speaker's awareness. Another instance is ‘lapses in monitoring of discourse’, which is characterised by Main as a loss of perceptual attention to the current environment when faced by a potentially distressing topic, leading to inappropriate statements. Again, Bowlby's technical term is not used by Main, but this is a recognisable instance of what Bowlby termed ‘defensive exclusion’.

The influence of Bowlby may have contributed to Main's usage of the terms ‘traumatic’ and ‘unresolved’ to refer to experiences of loss. In his book *Loss*, Bowlby characterised loss as a form of trauma. Indeed, the name of the first chapter is ‘The Trauma of Loss’. *Loss* contains a few other appearances of trauma discourse. For example, Bowlby spoke of ‘traumatic loss’ when discussing cases of mothers whose children died from choking or leukaemia (Bowlby, 1980: 163) and used the term ‘traumatic circumstances’ when referring to the death of a parent due to suicide (ibid.: 380). Bowlby did not use the term ‘unresolved’ in *Loss*, but it did appear several times in his early paper ‘Pathological Mourning and Childhood Mourning’ (1963). In this article, Bowlby extensively discussed the work of the psychoanalyst Helene Deutsch, who used the term ‘unresolved grief’ in the context of absent mourning and ‘unresolved experiences’ when referring to parental divorce (Deutsch and Jackson, 1937).

Yet Bowlby was not the only influence on the conceptualisation of unresolved states of mind. From Main's perspective, the development of the unresolved state of mind category was inductive.^20^ However, this induction can be considered historically in context of ideas circulating in her academic and cultural context. In the next section, we characterise how the assumptions about trauma in the development of the unresolved state of mind construct in the early 1980s can be placed in the context of contemporary discourses of trauma, in particular: the introduction of PTSD in the third edition of the Diagnostic and Statistical Manual of Mental Disorders (DSM-III) in 1980 and discourses about child abuse in the US in the 1960s–1980s.

### Contemporary discourses of trauma

#### Post-traumatic stress disorder in the DSM-III (1980)

One background influence on Main's attention to trauma may well have been the introduction of PTSD in the DSM-III (American Psychiatric Association, 1980). The introduction of PTSD marked a shift in attitude towards trauma in psychology and psychiatry. Previous conceptualisations of trauma assumed that some individuals had inherent weaknesses or biological predispositions causing traumatic symptoms. In contrast, PTSD implied that any person who had experienced an extremely distressing event could develop psychological and physiological symptoms, such as flashbacks, nightmares, and physiological arousal (Bracken, 2001; Fassin and Rechtman, 2009).

A general conceptual influence of PTSD on the unresolved state of mind construct may be the use of the term *trauma* to refer to experiences that are associated with lapses in reasoning and discourse in the Adult Attachment Interview. An early version of the lack of resolution of mourning scale included one reference to trauma: individuals with a high rating on the scale were believed to have experienced a ‘traumatic’ loss.^21^ The term *trauma* was more frequently mentioned in later editions of the coding system, for example, to refer to ‘traumatic events’, ‘traumatic experiences’, and ‘unresolved trauma other than loss’.^22^ Main and Hesse also referred to the DSM when introducing the unresolved state of mind classification in 1990: ‘The term “trauma” traditionally refers to experiences of intense fear, terror, or helplessness (see DSM-III-R) … which threaten an individual with psychological or behavioural disorganization’ (Main and Hesse, 1990: 162).

As with PTSD, the construct of an unresolved state of mind acknowledged a direct relation between a traumatic event and its manifestations: a distressing event would cause a certain behavioural response. However, events that were regarded as traumatic differed between the two constructs. In PTSD, traumatic events were defined as ‘unusual human experiences … [that] would be markedly stressing to almost anyone’, with a wide range of examples such as military combat, rape, and natural disasters, but also serious threat or harm to loved ones, such as relatives and friends (American Psychiatric Association, 1980). According to this definition of trauma, experiences of childhood physical or sexual abuse by attachment figures would count as traumatic events. The way traumatic events were conceptualised in PTSD may have influenced Main and colleagues’ decision to set qualifying thresholds for traumatic abuse in the unresolved state of mind classification, based on standardised definitions of which experiences would be considered overwhelmingly terrifying. A difference here, however, is that abuse could be coded as unresolved only if the perpetrator was an attachment figure, such as a parent. It is possible that this focus on abuse by attachment figures would make Main and colleagues’ theorising more easily accepted by the attachment field as relevant to attachment, as methods for assessing abuse were underdeveloped at the time.

Main did acknowledge that other events than physical and sexual by attachment figures could be potentially traumatic, such as sexual abuse by strangers or being witness to ‘extreme events’, such as a suicide attempt by a parent.^23^ These events could be considered traumatic according to the PTSD definition, but did not count towards a rating on the unresolved state of mind scale. A possible reason for not including these in the coding scale may have been that these experiences were not found in the interview transcripts of the development sample. But it may also be possible that Main and colleagues were unsure about whether such experiences were relevant to attachment.

A ‘simple bereavement’ was not listed as a traumatic event in PTSD, as it was considered a common experience (American Psychiatric Association, 1980). This is different from the way loss experiences were treated in the unresolved state of mind classification: there was no qualifying requirement that the loss of an important person, or the circumstances surrounding it, had to meet a certain threshold of ‘traumatic’ to be marked as unresolved. On the other hand, Main and Hesse (1990) did not consider all losses to be traumatic. This depended on individual perceptions and on conditions surrounding the loss, although Main and Hesse did not make explicit what such conditions could be. It may well be possible that conditions around loss were viewed as relatively unimportant by Main and colleagues, because the focus of the Adult Attachment Interview was not on the facts of the past but on how these were narrated. Indeed, Hesse has mentioned that

We do not try to establish whether the loss was in fact traumatic (we have no way to prove that). But rather, we assume that there is a high probability that it was traumatic, because these lapses [in reasoning or discourse] are associated with a psychodynamic propensity to enter into states that produce frightening/frightened behaviour and hence disorganised attachment in offspring.^24^

It may also be possible that, through the influence of Bowlby's work (1980), loss had already been established as a potentially traumatic experience having relevance to attachment, and Main and colleagues may not have felt a need to justify this in the Adult Attachment Interview coding system.

Another influence of PTSD on unresolved states of mind may have to do with the phenomenon of intrusions. A prominent symptom of PTSD was ‘sudden acting or feeling as if the traumatic event were recurring’ in which the individual may feel as if they are reliving the experience. The term ‘intrusive recollections’ was used to refer to these kinds of experiences (American Psychiatric Association, 1980). In the unresolved state of mind classification, a direct reference to intrusions can be found in the lapse that refers to visual-sensory intrusions, such as the following example in the unresolved abuse scale: ‘And then he became after me, and I’m running up the stairs, count ’em – one, two, three, four, bang! Duck around the door just it hit the wall near my head’.^25^ However, the PTSD symptom of intrusions may have influenced the conceptualisation of an unresolved state of mind more broadly than this lapse. In later writings, Main used the term ‘intrusion’ in attempts to explain possible underlying psychological mechanisms of the lapses in reasoning or discourse. For example, Main and Hesse proposed that the incoherent speech of adults classified as unresolved might be due to ‘partial intrusion of frightening, normally dissociated memories’ (Main and Hesse, 1992: 87). A major difference here, however, is that PTSD symptoms were mentioned by a patient or observed by a clinician, whereas in the Adult Attachment Interview, manifestations of such dissociative intrusions are inferred from ‘lapses’ in speech. Main and colleagues offered no evidence on whether these lapses are intrusions or other psychopathological symptoms. This question has not been examined by subsequent attachment researchers, who appeared to have taken the matter for granted on Main's authority, in the context of being trained for coding the Adult Attachment Interview.

Thus, the conceptualisation of unresolved states of mind as a form of trauma should be placed in the context of the introduction of PTSD in the DSM-III. But the unresolved state of mind construct clearly differs from PTSD in terms of the experiences that are regarded as traumatic, and how the psychological consequences of these events are inferred and assessed. A possible reason why Main diverged from the PTSD framework of trauma is that her account of trauma was influenced by both PTSD and psychodynamic theory, which was an important framework for both Bowlby and her graduate advisor Ainsworth (Main, 1999). Though not explicit in her published texts of the period, a possible influence on Main's conceptualisation of unresolved states of mind was the idea from psychodynamic theory of pathological mourning as a response to loss. The concept was frequently discussed by Bowlby (e.g. Bowlby, 1963, 1980), referencing psychoanalysts such as Freud and Klein. According to psychodynamic theory, pathological mourning could be manifested as defensive processes such as splitting, repression, and dissociation. These processes, often occurring outside awareness, could be elicited in psychotherapy. Main especially drew on the concept of splitting, which referred to the idea that a person's internal state could be divided in two parts: one part acknowledging the death of a loved one, and another part wishing that the loved one were still alive (Freud, 1927, cited in Bowlby, 1980). Main's interest in psychodynamic theory was only nascent in the 1980s during the formulation of the unresolved state of mind classification, but would be developed in two publications for the *Journal of the American Psychoanalytic Association* speculating on the psychodynamic basis of unresolved/disorganised states (Hesse and Main, 2000; Main, 2000).

#### The problematisation of child abuse in the United States (1960s–1980s)

Main and colleagues’ definition of child abuse in the Adult Attachment Interview was relatively narrow: only frightening experiences of physical or sexual abuse by an attachment figure could be marked as abuse. The coding manual listed three other experiences of qualifying abuse by attachment figures, which were suicide attempts in the presence of the child, threats to harm or kill the child, and expressions of bizarre and frightening behaviour in front of the child. Experiences that were merely distressing or upsetting, such as undeserved spankings that did not leave markings, were not coded as abusive. Coders of the Adult Attachment Interview were instructed to ‘probably plan to err on the side of exclusion’, ‘whatever the speaker's opinion’.^26^ This narrow definition of abuse has been criticised by subsequent attachment researchers, especially those with clinical training (George and Solomon, 1996; Levinson and Fonagy, 2004; Lyons-Ruth *et al*., 2003). However, Main and colleagues’ attention to only these kinds of child abuse can be placed in a wider social context.

The problematisation of child physical and sexual abuse in the US in the 1960s–1980s may have been a cultural discourse of trauma that influenced the conceptualisation of unresolved states of mind (Jenkins, 2004). Child abuse became recognised as a social problem in the United States from the early 1960s.^27^ Paediatricians used the term ‘battered child syndrome’ to refer to physical violence to children, usually by a family member, which was made visible through X-rays (e.g. broken bones). This was widely picked up by the media, and child abuse rapidly gained attention by the public, politicians, teachers, and social welfare professionals. From the early 1970s, there was also growing attention to the problem of child sexual abuse, raised by calls from feminist and humanitarian activist groups. These developments may have influenced Main and colleagues’ focus on experiences of physical and sexual abuse.

Interestingly, neglect had been regarded as a form of child abuse in American psychological discourse and wider culture since the 1960s. Yet neglect was not considered a potentially traumatic event by Main and colleagues in their unresolved abuse scale. Rather, neglect received its own coding scale in the Adult Attachment Interview manual, referring to parents who were physically available but uninvolved or psychologically inaccessible, but this scale did not contribute to assignment of the unresolved state of mind classification.^28^ A possible reason for Main not to consider neglect as a form of traumatic abuse is that these experiences were not reported by parents with disorganised infants in the development sample (Main and Hesse, 1990), and thus neglect did not become part of the theoretical framework of unresolved states of mind. Another factor may be that neglect is an omission of care rather than a locatable event, whereas unresolved states of mind are coded on the basis of discrete events. In addition, it is possible that Main did not regard neglect as a frightening experience. In the ‘Neglecting: Inaccessible when physically available’ coding scale, Main and colleagues defined neglect as ‘absence of interaction when potentially readily available to be present in the household’. Although Main and colleagues acknowledged that at high levels of parental neglect there could be a lack of connection between the parent and child, they considered that even children with highly psychologically or physically inaccessible parents ‘may feel more unnoticed than disliked, avoided and rejected’.^29^ Main has subsequently been criticised on this point by clinically trained attachment researchers, who have argued that the range of frightening parental behaviour should be expanded to include experiences of neglect (e.g. Lyons-Ruth and Block, 1996). Main and colleagues have not adapted the scale in response to these proposals.

Similarly, the unresolved abuse scale did not include emotional abuse (or psychological abuse; the terms are used interchangeably in the literature) as a potentially traumatic experience, despite this being a recognised form of maltreatment by the 1980s. Indeed Bowlby had been advocating for acknowledgement of emotional abuse and neglect as social problems already by the early 1970s.^30^ Nonetheless, one reason that Main and colleagues may have excluded emotional abuse was that in the early 1980s, a clear definition and theoretical framework of emotional abuse was lacking, causing practitioners as well as researchers to ‘stumble around in the dark’ (Garbarino, 1978). Another reason for excluding emotional abuse may be that emotionally abusive behaviours were already included in the scales for coding rejection and role-reversal in the Adult Attachment Interview, predicting infant insecure-avoidant and insecure-ambivalent attachment, and not disorganised attachment.

Since the 1980s, continuing theoretical and empirical attempts by developmental researchers have led towards improved operational definitions of emotional abuse as well as a growing body of evidence on the effects of emotional abuse and neglect on the development and well-being of children (e.g., Glaser, 2002; Thompson and Kaplan, 1996; see also Norman *et al*., 2012). Despite these developments, the definition of abuse in the unresolved abuse scale has not changed: it is still mainly focused on experiences of physical and sexual abuse.

### Obstacles to the integration of the unresolved state of mind with trauma research

Developments within the attachment field may have further contributed to the unresolved state of mind classification appearing as an authoritative construct representing trauma. An important factor may have been the strong theoretical connection between unresolved states of mind and infant disorganised attachment. In the late 1980s, child maltreatment was seen as the primary precursor of disorganised attachment in high-risk samples (e.g. Carlson *et al*., 1989). However, in general population samples, the prevalence of maltreatment was assumed to be lower than the rates of disorganised attachment (Main and Hesse, 1990). Main and colleagues introduced the unresolved state of mind category as another potential pathway leading to infant disorganisation, which served to explain the proportion of infant disorganised attachment in general population samples: ‘We underscore here that the infant's *D* Strange Situation response in low-risk samples such as ours is not normally an indication of maltreatment. Indeed, we will argue here for a quite different, although related, mechanism’ (ibid.: 165). Main and Hesse hypothesised that parents showing unresolved discourse in the interview were still frightened by memories of traumatic experiences such as loss. These parents would sometimes show frightened/frightening behaviours in the presence of their infant, such as unusual speech or movement patterns, which could lead to infant disorganised attachment behaviour. The idea that unresolved states of mind were associated with infant disorganised attachment, mediated by frightened/frightening parental behaviour, thus built on the underlying assumption that an unresolved state of mind was a construct related to fear and trauma. Indeed, Main and Hesse themselves assumed that parents with unresolved states of mind were ‘still-traumatised’ (ibid.: 163).

The theoretical link between unresolved states of mind and infant disorganised attachment was formulated alongside with early findings from the Berkeley sample: out of the 12 mothers identified with unresolved loss, 11 had infants classified as disorganised in the Strange Situation (Main and Hesse, 1990). This association was also found by subsequent studies, with the first few studies showing extremely high effect sizes (Ainsworth and Eichberg, 1991; Ward and Carlson, 1995). The link between unresolved states of mind and disorganised attachment has been less strong in later studies. However, the findings from the early studies may have led to the premature assumption among attachment researchers that unresolved states of mind represented trauma, and that this was ‘transmitted’ to the next generation (Verhage *et al*., 2016). In addition, later studies provided empirical evidence for the association of unresolved states of mind with frightened/frightening parental behaviour and infant disorganisation (Madigan *et al*., 2006; Schuengel, Bakermans-Kranenburg, and Van IJzendoorn, 1999), further adding to the theoretical meaning and nomological validity of the unresolved state of mind construct.

It seems that attachment researchers generally have taken for granted that an unresolved state of mind exists in the real world and that it represents a form of unresolved trauma.^31^ This may have hindered empirical inquiry into the relationship between the unresolved state of mind construct and scientific research on trauma, also by non-attachment researchers. Due in part, perhaps, to this lack of inquiry, the unresolved state of mind construct has not changed since its introduction, despite advances in knowledge about trauma from the wider field of developmental psychopathology research. As Stovall-McClough and Cloitre (2006: 219), two developmental psychologists, have observed, ‘Trauma theory and attachment theory have developed along relatively independent lines.’ Most strikingly, it is still unknown how the unresolved state of mind construct relates to standardised measures of trauma. A few researchers, mostly from the attachment field, have explored how unresolved states of mind may be related to PTSD (e.g. Harari *et al*., 2009; Nye *et al*., 2008; Turton *et al*., 2004). But studies so far have focused only on the unresolved state of mind classification as a whole, not considering potential associations between discrete manifestations of unresolved states of mind and trauma indicators from standardised measures.

One reason for the lack of interplay between the unresolved state of mind construct and wider trauma discourses may have been confusion about what it is that an unresolved state of mind actually measures, to those both inside and outside the attachment field. Main and colleagues’ explanations of the psychological mechanisms behind unresolved discourse have been somewhat ambiguous. Over the years, the lapses in reasoning or discourse have been suggested to indicate ‘continual mental disorganization and disorientation’ (Main and Hesse, 1990: 168), ‘partial intrusion of frightening, normally dissociated memories’ (Main and Hesse, 1992: 87), ‘lapses in working memory’ (ibid.: 96), and ‘micro-dissociative states’ (Main and Morgan, 1996: 126). Main and Hesse did not pursue to develop these proposals into testable hypotheses and left the task to others to integrate these ideas with other scientific studies of trauma phenomena. An exception is the study by Hesse and Van IJzendoorn (1999), exploring the association between absorption (an aspect of dissociation) and unresolved states of mind. Subsequent researchers have attempted to address some of Main and colleagues’ ideas about the underlying psychological mechanisms of unresolved discourse, for example, by examining the role of dissociative experiences (e.g. Schuengel, Bakermans-Kranenburg, and Van IJzendoorn, 1999) and differences in stress-sensitive brain regions (e.g. Van Hoof *et al*., 2019) in adults classified with an unresolved state of mind.

The lack of dynamic interplay between the unresolved state of mind construct and other areas of knowledge about trauma may be conceptualised as an effect of the relative autonomy of the attachment field (Bourdieu, 1975). As Duschinsky (2020) has observed, the field of attachment research is somewhat insular: it is a part of developmental science, but also in a way detached from it. Another reason for the lack of interplay of the unresolved state of mind classification with other disciplines may be that Main and her colleagues did not put the Adult Attachment Interview coding system into wider circulation. In 1986, Main had completed a book in which she described various measures developed for the Berkeley Social Development Study, including descriptions of the Adult Attachment Interview coding system. However, this book remained unpublished.^32^ The coding manual was, and is still, provided to and only to be used by participants of intensive two-week training institutes. Learning to code the Adult Attachment Interview is an expensive and laborious process. Besides taking part in the training institutes, there is no other way of gaining detailed knowledge about unresolved states of mind. In this context, we are grateful that Main and colleagues have given us copies of these manuals for use in our historical research. Main and colleagues have sought to control the circulation of knowledge of how the Adult Attachment Interview is coded in order to reduce the potential risks of unlicensed applications by untrained researchers and of demand characteristics for participants. Yet, from a historical perspective, we would observe that publication of the 1986 book and/or the Adult Attachment Interview coding manual might have helped bring her ideas on measurement into greater dialogue with the wider field of developmental science.

## Conclusions

This article traced the emergence of the unresolved state of mind classification, placing Main's conceptualisation of an unresolved state of mind in wider disciplinary and social context. We observed that there are multiple assumptions of trauma embedded in the construct of an unresolved state of mind: (a) bereavement is functionally identical to abuse as a kind of trauma when not psychologically reconciled; (b) traumatic abuse experiences are defined by child physical and sexual abuse by attachment figures; and (c) lapses in adult discourse about unresolved bereavement or abuse experiences are underpinned by the same process as disruptions of attachment behaviour shown by young children. A proximal influence on all three assumptions was Bowlby, who had made bereavement and trauma his central focus in *Loss* (1980) and conceptualised segregated systems and defensive exclusion following loss as mechanisms that could disrupt the coherence of behaviour and speech in children and adults. Further, we related the development of the unresolved state of mind construct to broader contemporary trauma discourses, in particular the introduction of PTSD in the DSM-III in 1980 and discourses about child abuse in the US in the 1960s–1980s. We have seen that, despite advances in knowledge from wider disciplines of trauma, the construct of an unresolved state of mind has remained unchanged. For example, the definition of child abuse in the unresolved state of mind classification is still focused on mainly child physical and sexual abuse, and is therefore relatively narrow compared to contemporary definitions of child abuse that include neglect and emotional abuse.

While attachment researchers have generally taken for granted that an unresolved state of mind represents a form of unresolved trauma, there has been a lack of interaction between the unresolved state of mind construct and other areas of knowledge about trauma. The unresolved state of mind classification was developed within the context of wider scientific and social debates about trauma but has also remained detached from it. This may be an effect of the relative autonomy of the attachment field, as well as confusion – to both those within and those outside the attachment field – about what it is that the construct of an unresolved state of mind actually represents. The decision by Main and colleagues to limit circulation of the Adult Attachment Interview coding manual to those who attend the two-week training course may have contributed to the lack of interplay of unresolved states of mind with wider trauma disciplines.

Our article contributes to the history of attachment research by documenting the development of Main and colleagues’ measure of unresolved loss and abuse. We have shown that Bowlby's work on trauma and loss was a fundamental influence on the conceptualisation of unresolved states of mind, and investigated broader disciplinary influences that may have helped shape the construct. This article also contributes to the historiography of conceptualisations of trauma, by describing a psychological measure characterised by an intriguing amalgamation of loss with trauma, which became a construct representing unresolved trauma and gained status within the relatively isolated area of attachment research.

## Notes

The authors thank Marian Bakermans-Kranenburg, Marinus van IJzendoorn, and Everett Waters for their helpful feedback on the article.

1. M. Main, E. DeMoss, and E. Hesse, *Unresolved (Disorganized/Disoriented) States of Mind With Respect to Experiences of Loss*, 1991/1994, unpublished manuscript, Department of Psychology, University of California, Berkeley.

2. M. Main, *Disorganized Attachment in Infancy, Childhood, and Adulthood: An Introduction to the Phenomena*, 1999, unpublished manuscript, Mary Main and Erik Hesse personal archive.

3. C. George, N. Kaplan, R. Goldwyn, and M. Main ‘Attachment Interview for Parents’, 1982–3, Wellcome Trust, London, John Bowlby Archive (hereafter ‘Bowlby Archive’), PP/Bow/B.3/35-6.

4. C. George, N. Kaplan, and M. Main, *Adult Attachment Interview* (3rd ed.), 1996, unpublished manuscript, Department of Psychology, University of California, Berkeley.

5. M. Main, *Behaviour and the Development of Representational Models of Attachment: Five Methods of Assessment*, 1986, unpublished manuscript, Mary Main and Erik Hesse personal archive.

6. M. Main, ‘Lack of Resolution of Mourning’, 1987, Bowlby Archive, PP/Bow/B.3/35-6. In this scale, the use of ‘attachment figures’ was not limited to parents or family members. Main and colleagues noted that ‘loss of any family member is significant, as is loss of anyone with whom the subject lived in childhood (or who lived in the family home). Some persons were very fond of and lost a non-family member. Note whether these persons seemed to serve as attachment figures for the subject.’

7. Ibid.

8. Ibid.

 9. Ibid.

10. Ibid.

11. A discrete version of the unresolved abuse coding scale was finished in 1991. The latest version of the Adult Attachment Interview coding system (version 7.2, 2003) still contains the 1991 version.

12. M. Main, R. Goldwyn, and E. Hesse, *Adult Attachment Scoring and Classification Systems*, 2003 p. 149 (emphasis in original), unpublished manuscript, Department of Psychology, University of California, Berkeley.

13. M. Main, R. Goldwyn, and E. Hesse, *Adult Attachment Scoring and Classification Systems*, 2003, unpublished manuscript, Department of Psychology, University of California, Berkeley.

14. J. Bowlby, Letter to Tirril Harris, 17 September 1985, Bowlby Archive, PP/Bow/J.9/94.

15. J. Bowlby, ‘Defences That Follow Loss: Causation and Function’, 1962, Bowlby Archive, PP/Bow/D.3/78.

16. Ibid.

17. Ibid.

18. J. Bowlby, ‘Notes Following Discussion With Mary Main in March 1978 About the Draft of Vol. 3 Loss’, 1978, Bowlby Archive, PP/Bow/H.78.

19. M. Main, Letter to Bowlby, 12 September 1986, Bowlby Archive, PP/Bow/B.3/35.

20. M. Main, Personal communication, 15 May 2021.

21. Main, ‘Lack of Resolution of Mourning’.

22. M. Main and R. Goldwyn, *Adult Attachment Scoring and Classification Systems*, 1991, unpublished manuscript, Department of Psychology, University of California, Berkeley.

23. M. Main, *Unresolved Trauma Other Than Loss: Abusive, Threatening or Very Frightening Behavior of an Attachment Figure*, 1991, unpublished manuscript, Department of Psychology, University of California, Berkeley.

24. E. Hesse, Personal communication, 13 May 2021.

25. M. Main, R. Goldwyn, and E. Hesse, ‘*Adult Attachment Scoring and Classification Systems*’, 2003, p. 153, unpublished manuscript, Department of Psychology, University of California, Berkeley.

26. Ibid.: 147.

27. This refers to the modern construct and exact term of ‘child abuse’. Before the 1960s, similar experiences were referred to as ‘cruelty to children’. Differences between the two concepts are described in Hacking (1998).

28. M. Main, R. Goldwyn, and E. Hesse, ‘*Adult Attachment Scoring and Classification Systems*’, 2003, p. 35, unpublished manuscript, Department of Psychology, University of California, Berkeley.

29. Ibid.

30. J. Bowlby, ‘Evidence Presented on 6/4/1971 to the Committee on the Adoption of Children, Home Office and Department of Health and Social Security’, 1971, The National Archives, BN 29/2340.

31. An exception is the empirical work by Roisman and colleagues. Based on taxometric findings, they have suggested that ‘there may be no empirical distinction between indicators of preoccupation and unresolved discourse’ (Haltigan *et al*., 2014; Roisman *et al*., 2007).

32. M. Main, *Behaviour and the Development of Representational Models of Attachment: Five Methods of Assessment*, 1986, unpublished manuscript, Mary Main and Erik Hesse personal archive.
